# Using Natural Selection to Explore the Adaptive Potential of *Chlamydomonas reinhardtii*

**DOI:** 10.1371/journal.pone.0092533

**Published:** 2014-03-21

**Authors:** Marie-Mathilde Perrineau, Jeferson Gross, Ehud Zelzion, Dana C. Price, Orly Levitan, Jeffrey Boyd, Debashish Bhattacharya

**Affiliations:** 1 Department of Ecology, Evolution and Natural Resources, Rutgers University, New Brunswick, New Jersey, United States of America; 2 Institute of Marine and Coastal Science, Rutgers University, New Brunswick, New Jersey, United States of America; 3 Department of Biochemistry and Microbiology, Rutgers University, New Brunswick, New Jersey, United States of America; Michigan State University, United States of America

## Abstract

Improving feedstock is critical to facilitate the commercial utilization of algae, in particular in open pond systems where, due to the presence of competitors and pests, high algal growth rates and stress tolerance are beneficial. Here we raised laboratory cultures of the model alga *Chlamydomonas reinhardtii* under serial dilution to explore the potential of crop improvement using natural selection. The alga was evolved for 1,880 generations in liquid medium under continuous light (EL population). At the end of the experiment, EL cells had a growth rate that was 35% greater than the progenitor population (PL). The removal of acetate from the medium demonstrated that EL growth enhancement largely relied on efficient usage of this organic carbon source. Genome re-sequencing uncovered 1,937 polymorphic DNA regions in the EL population with 149 single nucleotide polymorphisms resulting in amino acid substitutions. Transcriptome analysis showed, in the EL population, significant up regulation of genes involved in protein synthesis, the cell cycle and cellular respiration, whereas the DNA repair pathway and photosynthesis were down regulated. Like other algae, EL cells accumulated neutral lipids under nitrogen depletion. Our work demonstrates transcriptome and genome-wide impacts of natural selection on algal cells and points to a useful strategy for strain improvement.

## Introduction

Growing global human population size places significant demands on hydrocarbon resources [1]. This has resulted in a surge in biofuel research to provide alternative energy sources that are renewable and carbon-neutral with respect to greenhouse gases. Algae have been of particular interest because of their high productivity [2]–[4] and because they do not usually compete for arable land and potable water. These considerations have led to the search for fast growing, stress resistant, lipid-producing algae (e.g., diatoms, green algae, chrysophytes; [5]). Target taxa would normally be subject to crop improvement by breeding (as routinely done for crop plants; e.g., [6], [7]), however this approach requires knowledge about the sexual cycle that is lacking for many algae. Therefore, absent access to sexual recombination to develop hybrids and public misgivings about cultivating genetically engineered algae in open ponds [8], an alternative approach to strain improvement is experimental evolution [9]–[13]. This approach relies on the natural capacity of microbes to adapt rapidly to changing environmental conditions (e.g., ocean acidification; [14]) and can lead to the generation of novel traits of interest. However, unlike culture perturbations that focus on specific pathways such as the carbon-concentrating mechanism [15], [16] or the response to sulfur deprivation [17], serial transfer of eukaryotes placed under selective regimes over hundreds of generations may open up a “Pandora's box” of genetic variation within populations [18]. This variation would be reflected in the accumulation of DNA mutations, gene expression changes, and epigenetic modification [19] that impact a variety of metabolic pathways [20]. Here we tested the utility of strain improvement vis-à-vis long-term selection [11], [14], [20] using as inoculum a single colony of the cell wall-deficient mutant *C. reinhardtii* strain CC-503 (cw92 mt+) that has a sequenced genome [21].

## Materials and Methods

### Strain and culture conditions

*Chlamydomonas reinhardtii* strain CC-503 cw92 mt+, used by the Joint Genome Institute for genome sequencing, was cultivated in axenic conditions at 25°C under continuous light (100 μE/m^2^/s) on a rotary shaker at 100 rpm (Innova 43, New Brunswick Eppendorf). A single colony was used to make a starter culture referred to as the “Progenitor” and was subcultured in liquid tris-acetate phosphate medium (TAP; [22]). *C. reinhardtii* subculturing was done during the exponential growth phase (optical density [OD] between 0.4 and 0.7 at λ = 675 nm) by the dilution of 1 ml of the culture into 100 ml of TAP medium (every 1–2 days). The subculturing (i.e., serial transfers) was done for 17 months (from February 2011 until June 2012). Progenitor light (PL) refers to the *C. reinhardtii* cells obtained after the first subculturing steps in TAP medium and under continuous light. The evolved light (EL) culture corresponds to *C. reinhardtii* cells evolved for 1,880 generations (283 subcultures). To compare the growth rate between the two *C. reinhardtii* cultures (PL and EL), a growth curve was done during the exponential phase with each culture at an initial concentration of ∼5×10^5^ cells/ml (O.D. ∼0.2). The cultures were done in triplicate and the cells counted using a Beckman Multisizer 3 Coulter Counter every 12 hours over 5 days.

### Microscopy

The effects of experimental evolution on neutral lipid content were visualized using laser scanning confocal microscopy. The PL and EL *C. reinhardtii* cultures, after 48 h in their respective TAP medium with or without nitrogen (7 mM NH_4_Cl), were stained with the nonpolar lipid fluorophore Bodipy 493/503 (Molecular Probes; [23]). To concentrate the cells, 2 ml of each culture was centrifuged at 13,000 rpm for 1 min. The supernatant was removed, and 200 μl of this liquid was used to resuspend the cell pellet. The cells were stained with 8 μM Bodipy 493/503 for at least 5 min in the dark. To immobilize cells, 25 μl of 1% agarose was spread and fixed on a slide then 25 μl of stained cells were added and covered with a coverslip.

Images were acquired using a Zeiss LSM 710 Confocal Microscope (Carl Zeiss MicroImaging GmbH) built on an AXIO Observer Z1 inverted microscope as the imaging platform, with a 63X water objective. The excitation laser was 633 nm for chlorophyll autofluorescence and detected using a 647/721 nm spectral detection channel (red fluorescence). The Bodipy 493/503 was excited with a 488 nm laser and the fluorescence was detected using the spectral detection channel (493/589, green fluorescence). Image acquisition and processing were done using the ZEN 2010 software (Zeiss Efficient Navigation).

### DNA and RNA extraction and processing

For DNA and RNA extraction, the cells (under continuous light) were harvested during the exponential phase using centrifugation at 4,000 rpm for 1 min and then immediately stored in liquid nitrogen. DNA extraction was done using the DNeasy Plant Mini Kit (Qiagen) and total RNA was extracted using the RNeasy Mini Kit (Qiagen). Both of these procedures utilized ca. 100 mg of fresh tissue and were processed according to the manufacturer's instructions. For the RNA extraction, three independent cultures were generated for each treatment. DNA and RNA concentrations were determined using a NanoDrop 2000c Spectrophotometer (ThermoScientific). DNA contaminant from RNA preparations was removed using the TURBO DNA-free DNase kit (Ambion). Single stranded cDNA was synthesized from 500 ng of total RNA using the Super script III First-Stand Synthesis System using random hexamers (Invitrogen).

### Illumina library construction

DNA and cDNA libraries were construction using the Nextera DNA Sample Preparation Kit (Illumina) and the Nextera Index Kit (Illumina). The *C. reinhardtii* libraries were sequenced using the GAIIx Genome Analyzer (Illumina) and/or the MiSeq Personal Sequencer (Illumina) (Table S1). The RNA-seq and genome data are available at the NCBI Sequence Read Archive (SRA) under accession code SRP037997.

### Quantitative polymerase chain reaction

To confirm the RNA-seq results, the expression level of several genes under the two different culture conditions (PL and EL) was measured using quantitative PCR (qPCR). The genes and the inteIllumina l reference (mitochondrial cytochrome c oxidase subunit) were chosen based on the transcriptomic analysis (primers listed in Table S2). qPCR was performed with the ABI StepOne Plus real-time PCR System (Applied Biosystems, USA) using the KAPA SYBR® FAST qPCR Kit Master Mix ABI Prism, according to the manufacturer's instructions. The real-time PCR cycle was 95°C for 3 min followed by 40 cycles at: 95°C, 3 sec and 60°C, 30 sec. A dissociation curve analysis of the amplification products was performed at the end of each PCR reaction to confirm there was a single PCR product. Triplicate qPCRs were performed for each sample. The ΔΔCT method [24] was used to measure the relative fold quantification.

### Genome SNP/indel analysis

For the SNP/indel analysis, all of the Illumina genomic reads from the PL and EL populations were trimmed of adaptors and low quality nucleotides. These data were then mapped to the *C. reinhardtii* reference genome (version 5.3.1; http://www.phytozome.net/chlamy) using the CLC Genomic Workbench. The cut-offs used for this analysis were ≥85% of the sequence read matching to the reference genome with ≥90% identity over the matched region. The mapping files were analyzed using the CLC Genomic Workbench quality-based variant detection tool. SNPs/indels were called only if they had ≥20X coverage with the genome reads, a minimum Phred-like quality score of 30 (i.e., ≥99.99% accuracy) at the position of the SNP or insertion, and an average quality score >20 (i.e., ≥99% accuracy) of the flanking 5 bp around the variable nucleotide(s). To identify polymorphisms specific to EL, the variable positions were screened against SNPs/indels identified in the PL population.

### Transcriptome analysis

The RNA-seq reads from the PL and EL populations (each done in triplicate) were trimmed to remove sequencing adaptors and low quality nucleotides. The trimmed reads were then aligned to the annotated *C. reinhardtii* reference genome using CLC Genomics Workbench with a length fraction cutoff set to ≥85% of the sequence read matching to the reference genome with ≥90% identity over the matched region. Only reads that mapped uniquely to reference transcripts were counted and used for differential expression analysis. For the process of calling differentially expressed genes, the read count table was used as input to DESeq, the R/bioconductor package that is based on the negative binomial distribution [25]. The p-values of the overall test for each gene were adjusted for the false discovery rate (FDR) control at 5% [26]. To identify functions impacted by the experimental evolution experiment, these data were exported to Excel spreadsheets and significantly differentially expressed genes were used as input to the KEGG metabolic pathway-mapping tool (http://www.genome.jp/kegg/). Up and down-regulated genes were colored differently in the pathway outputs according to the KEGG instructions.

## Results

### Comparison of growth rates of progenitor and evolved cell populations

**Figure 1:**
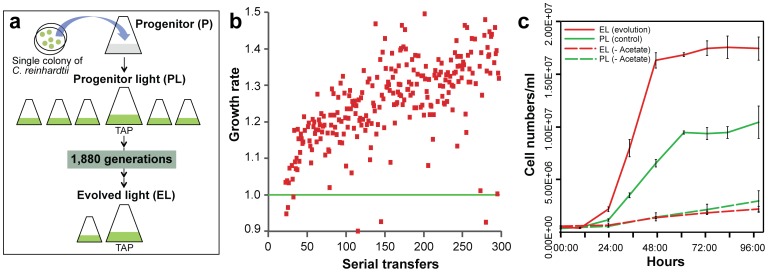
Growth rate of an evolved population of *C. reinhardti*. (A) Design of the experimental approach used to generate a rapidly growing *C. reinhardtii* CC-503 cw92 mt+ population. (B) Results after 283 transfers (1,880 generations) of cells grown under continuous light in liquid TAP medium. At the end of the experiment, the evolved *C. reinhardtii* population grows ca. 35% faster than the progenitor strain. The progenitor growth rate is indicated with the solid green line. (C) Comparison of growth rates of the progenitor (PL, green line) and one population of the evolved (EL, red line) *C. reinhardtii* cells grown in TAP medium. The growth rate of these populations when raised in acetate-free (TAP) medium is shown with the dashed green line for PL and the dashed red line for EL. These curves are derived from three independent culture replicates.

**Figure 2:**
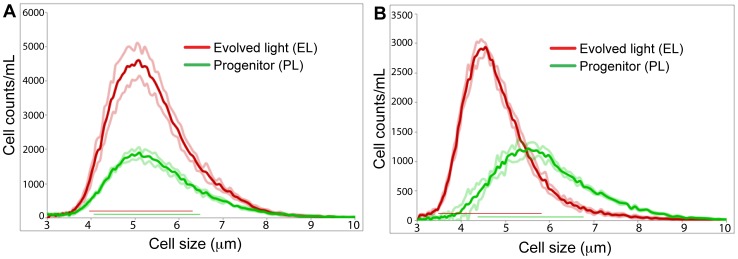
The distribution of cell sizes of exponential (A) and stationary phase (B) cultures of the PL (green line) and EL (red line) *C. reinhardtii* populations used in our analysis. The size at one standard deviation from the mean for each cell population is shown with the light colored lines. Although a broad range of cell sizes is present in each culture, the stationary phase EL population is on average ca. 1 μm smaller than the PL cells.

Our experimental design (Fig. 1A) involved propagating *C. reinhardtii* cells through vegetative growth in batch cultures on a regime of 1∶100 serial transfers. Adaptation in these evolved light (EL) cells was implied by an increase in growth rate when compared to the progenitor (PL) cells (Fig. 1B). After 1,880 generations (283 serial transfers), one culture (evolved light; EL) was chosen for genome analysis. This population had a growth rate that was ca. 35% greater than the PL population, a result that was validated by a comparison of PL and EL growth curves in triplicate cultures (Fig. 1C). Analysis of cell size showed surprisingly that in exponential phase PL (5.27±1.21 μm [standard deviation]) and EL (5.13±1.18 μm) cells were the same size despite clear differences in the growth rate. However, during stationary phase the EL cells were ca. 1 μm smaller in diameter (4.65±1.17 μm vs. 5.67±1.20 μm; Fig. 2). When acetate was removed from the culture medium, the PL and EL cells had significantly lower but equal growth rates (Fig. 1C).

### Analysis of DNA differences between Progenitor and Evolved cell populations

To determine whether the EL population had accumulated SNPs or indels (of any length), DNA from the PL and EL populations was sequenced and compared to the *C. reinhardtii* reference genome, following a filtering step to remove low quality reads and regions of low coverage. The average coverage was 24.10x and 42.71x with 93% and 92% of the genome covered in the PL and EL populations, respectively. To get a more accurate reading of coverage variation across the re-sequenced genomes, we sorted the genome mapping read-by-read and calculated the percentage coverage for each library per site that was ≥20x. These values are as follows: PL – 60.5% (67,287,991 bases), EL – 63.5% (70,610,468 bases).

We found 7,133 SNPs/indels in the EL population when compared to the reference genome. To identify polymorphisms specific to EL, the variable positions were screened against SNPs/indels identified in PL, which reduced the EL number to 1,937 (Table S3). Given that the nuclear genome was partially covered (i.e., accounting for 67.3 Mbp at ≥20x of the complete nuclear genome of size 111.10 Mbp) with 1,880 generations of evolution in our 100 mL cultures, the mutation rate for the EL population was estimated to be 1.53×10^−8^/base/generation (i.e., ca. 1.7 mutations per generation of 100 mL of cells). If we consider only the 1,782 single base SNPs/indels, the mutation rate was estimated to be 1.41×10^−8^/base/generation. The read mapping analysis indicated that the main types of substitutions among the 1,782 single base SNPs/indels were the transitions G:C→A:T, which accounted for 30.1% versus 22.0% for A:T→G:C (Table S3).

On the 1,937 SNPs/indels specific to the EL population, 914 (47%) and 1,023 (53%) occurred, respectively, in intergenic and genic regions. In the latter category 469 SNPs/indels were in introns, 313 in untranslated regions (UTRs), and 241 in coding regions. The 313 SNPs/indels in UTR regions were distributed among 170 genes. Of the 241 coding region changes, 149 gave rise to non-synonymous substitutions in 83 genes, and 92 gave rise to synonymous substitutions in 55 genes (Table S3). The locations of SNPs/indels in the genome was significantly different from the expected distribution based in the *C. reinhardtii* genome of 3∶7∶10 of exons:introns:intergenic regions [27], (χ2 test; df = 2, p = 2.7×10^−7^).

Only two genes containing SNPs had an existing annotation and were also significantly differentially expressed (see below). The first gene, a reverse transcriptase associated with a non-LTR retrotransposon (Cre01.g006700.t1.21, 1 non-synonymous SNP) was up regulated at Log_2_ fold change = 4.62 when comparing the PL-EL populations. The second, a histidine kinase rhodopsin (Cre07.g329900.t1.3, 2 non-synonymous and 5 synonymous SNPs) was significantly differentially expressed at Log_2_ fold change = 1.30.

### Transcriptome analysis of progenitor and evolved cell populations

**Figure 3:**
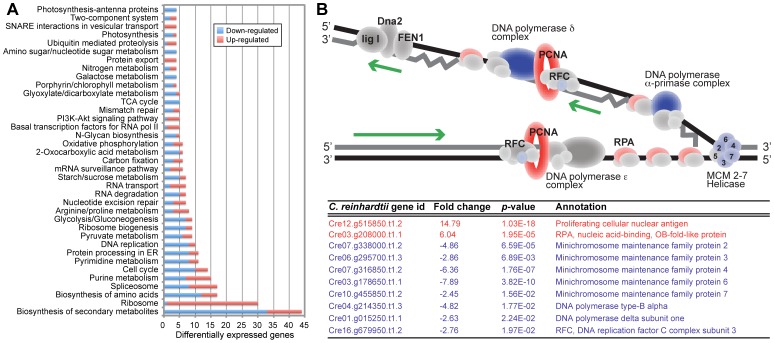
Transcriptome analysis of the EL population. (A) The number of significantly differentially expressed genes (not all genes) in the PL-EL comparison of triplicate RNA-seq samples. Only KEGG categories with ≥4 genes are shown. Significantly up and down-regulated genes are shown with the red and blue bars, respectively. The gray boxes include categories with ≥4 genes. (B) Schematic model of DNA replication (based on the KEGG model) showing the gene expression patterns when comparing the PL-EL populations. All protein components that do not show a significant expression difference are shown in gray, proteins that are significantly up regulated are shown in red, and proteins that are significantly down regulated are shown in blue. The Table below the model shows the results of the statistical analysis of gene expression for the significantly differentially expressed proteins in this complex.

**Figure 4:**
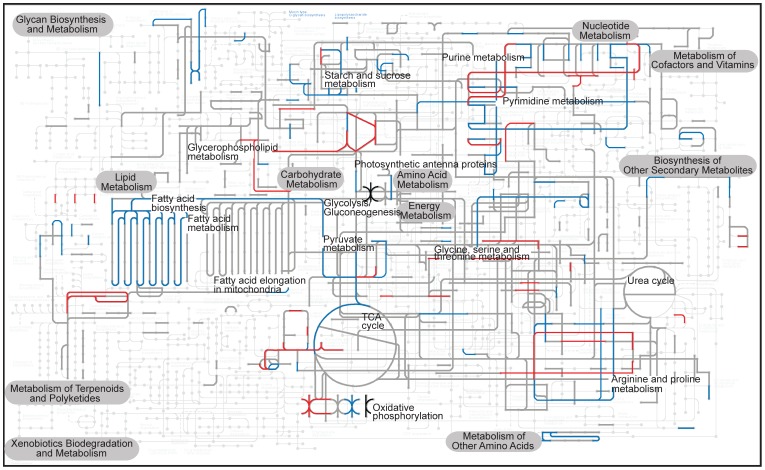
The distribution of significantly up and down-regulated genes in the PL-EL comparison when mapped on the KEGG metabolic pathway. The gray lines indicate the background RNA-seq data in this comparison, whereas the red and blue lines show genes that were significantly up and down-regulated, respectively. The black lines indicate genes that show both types of effects, due to the existence of gene paralogs with differential expression.

Transcriptome (Illumina RNA-seq) analysis using triplicate cultures of PL and EL cells harvested during exponential growth showed a consistent signal from each sample (Fig. S1A). Using the PL population as the control condition, analysis of Log_2_ fold changes in gene expression values showed 1,719 genes to be significantly up or down-regulated at *p*-value <0.05 (adjusted with the FDR correction [26]; Fig. S1B and Table S4). Consistent with previous results (e.g., [15], [28]) our differential expression results from RNA-seq were confirmed using quantitative PCR (qPCR; Fig. S2 and Table S2). To determine the number of differentially expressed genes in different Kyoto Encyclopedia of Genes and Genomes (KEGG; http://www.genome.jp/kegg/) categories we analyzed all KEGG categories with ≥4 genes (Fig. 3A). Moreover, we analyzed the distribution of up and down-regulated genes in the PL-EL comparison using the global KEGG metabolic pathways (Fig. 4) with a focus on several target pathways (see below).

The greatest number of significantly up-regulated genes encoded ribosomal proteins (Fig. 3A) with 29/30 proteins identified in this category (Fig. S3), proteins involved in RNA-transport to facilitate gene expression and support translation (5/7 differentially expressed gene functions), the translation initiation factor subunits eIF1, eIF3, eIF5 and eIF2B, two subunits of RNA polymerase (i.e., DNA-directed RNA polymerase II subunit RPB11 [mRNA precursor synthesis], and DNA-directed RNA polymerase III subunit RPC10 [small RNAs such as 5S rRNA and tRNA synthesis]) (Table S4). Several cell cycle genes were also up-regulated (e.g., S-phase kinase-associated protein [SCF], cyclin A [CycA], 14-3-3 protein, proliferating cell nuclear antigen [PCNA] 15-fold, replication factor 1 [RPA] 6-fold) (Fig. S4). We also found significant up-regulation of cytosolic acetyl-CoA synthetase (9-fold); some components of the respiratory complexes: *NUOB12* (NADH:ubiquinone oxidoreductase [2-fold]), *COX90* ([cytochrome c oxidase subunit 90 [2-fold]), *PET191* (cytochrome c oxidase assembly protein [8-fold]), and *QCR8, QCR9* (subunits of ubiquinol:cytochrome C oxidoreductase [5-fold and 4-fold, respectively]); some components of the mitochondrial Fe-S cluster biogenesis (e.g., iron-sulfur cluster assembly proteins *ISC1* [17-fold] and *ISU1* [2-fold]) and iron transport (*FTR1* [8-fold]) and the mitochondrial ROS metabolism (*GPX3*, *GPX5*, *TRXo*).

In the EL population, several pathways were significantly down regulated. These included the DNA replication pathway (8/10 gene functions), the pathway for the biosynthesis of secondary metabolites (33/44 gene functions), and photosynthesis (e.g., 4/4 antenna proteins). For the DNA replication pathway, the 5/6 subunits mini-chromosome maintenance (MCM) complex was significantly down regulated (Fig. 3B). For the biosynthesis of secondary metabolites, the fatty acid biosynthesis, metabolism, and elongation pathways were significantly down-regulated (Fig. S5) in the EL population (i.e., major targets include S-malonyltransferase [*fabD*] and 3-oxoacyl-[acyl-carrier-protein] synthase III [*fabH*]).

To address the possibility that the difference in library size between the PL and EL RNA-seq runs (Table S1) significantly impacted the differential gene expression analysis, we reduced the number of mapped reads per gene of the EL library using two different methods and re-ran DESeq. In the first method, we reduced (normalized) the number of mapped read counts per gene in the 3 EL replicates by dividing them by 5. In the second method, the reads in each PL replicate library were randomly sampled so that the sum of all mapped reads in each replicate matched the PL library sizes (1.9 M, 2.4 M and 1.3 M). Analysis of the reduced datasets showed a ∼20% decrease in the total number of differentially expressed genes; i.e., 1378 and 1391 genes in the samples divided by 5 and by using random sampling, respectively. However, out of the top 500 statistically significant differentially expressed genes in the original dataset 460 (92%) and 434 (87%) genes were present in the samples that were divided by 5 and in the random sampling approach, respectively. Minor differences were found in the differentially expressed ribosomal proteins (from 29/30 up-regulated genes in the original dataset to 26/27 in the divided by 5 and to 28/28 in the random sampling) and in the DNA repair pathway (from 8/10 down-regulated genes in the original dataset to 7/9 in the divided by 5 method and to 6/8 in the random sampling method). These results demonstrate that the differences in coverage did not significantly impact the major findings presented in our paper.

## Discussion

### Changes in *C. reinhardtii* growth rate and cell size

After 283 serial transfers in liquid TAP medium under continuous light (ca. 1,880 generations) the evolved *C. reinhardtii* population had a doubling time that was ca. 35% lower than the progenitor population. This relatively higher, evolved growth rate, of interest for industrial applications [4], was achieved within 100 serial transfers (ca. 6 months). An inverse relationship between growth rate and cell size is expected because smaller average cell size (i.e., a larger surface to volume ratio) presumably confers a greater capacity to acquire nutrients to support the increased growth rate [29], [30]. Surprisingly, the PL and EL cells did not show such a trend with no significant cell size difference during the exponential growth phase (Fig. 2). The elevated growth rate in the EL population in TAP medium is most likely explained by more efficient uptake and metabolism of acetate [11], supported by the observation that PL and EL cells had significantly lower but equal growth rates in the absence of this organic carbon source. The continuous light conditions used here could also play a role in the change in cell size and increase in growth rate [31] observed during stationary and exponential phases, respectively.

### Changes in the *C. reinhardtii* genome

In this study, we compared the genome of two *C. reinhardtii* populations following 17 months of serial transfer under a regime of continuous light and the presence of an organic carbon source. We estimated the mutation rate to be 1.53×10^−8^/base/generation for the EL population, which is 47–226 times higher than previous estimates made for this species (3.23×10^−10^ and 6.76×10^−11^) but comparable to higher plants such as *A. thaliana* (5.9×10^−9^, Table S3) [27], [32]. This result is not surprising if we take into account that the DNA repair pathway was down regulated in the evolved *C. reinhardtii* population (see discussion on the gene level expression), possibly leading to a higher mutation rate than has been previously estimated for this species. Among the 1,782 single base SNPs/indels, the mutations favored transitions G:C→A:T (30.1% and 22.0% for A:T→G:C, Table S3) as has already been shown in previous studies [27], [32] and consistent with an elevated GC-content of 61.74% in the *C. reinhardtii* nuclear genome.

The location of SNPS/Indels detected in the *C. reinhardtii* genome of the EL population was 47.2% in intergenic regions, 12.4% in exons, 24.2% in introns and 16.2% in UTRs (Table S3). This distribution is significantly different from random expectations (p<0.05) and departs from previous studies that were based on a smaller number of SNPs/indels being found due to the low mutation rate in this alga [27], [32].

The analysis of DNA differences between the *C. reinhardtii* PL and EL cell populations suggested that 83 genes contain a non-synonymous substitution. Of these, the reverse transcriptase associated with a non-LTR retrotransposon (Cre01.g006700.t1.21) that was significantly up regulated in the EL population likely indicates a stress response in these cells [33]. The other differentially expressed (up regulated) gene encodes a histidine kinase with a rhodopsin domain (Cre07.g329900.t1.3) at the N-terminus. This protein is related to the recently described light-activated chromoproteins in *C. reinhardtii* that mediate signaling processes [34]. Therefore the SNPs in this gene may have functional consequences for EL cells. Apart from these two examples of SNPs associated with genes showing expression differences in the EL population, most of the SNPs/indels we identified are likely to reflect genetic drift due to the serial transfer regime. We also expect that non-coding regions would be the primary targets for DNA sequence changes in the EL cells with potential impacts on epigenetic changes (e.g., micro RNA loci) regulating gene expression. For these reasons, and due to the inability using Illumina short-read data to assemble with high confidence non-coding, repeated regions in the highly complex *C. reinhardtii* genome, we studied the gene expression data for clues to explain the EL phenotype.

### Changes in the *C. reinhardtii* transcriptome

This study is the first to analyze global gene expression differences in a green alga as a result of long-term experimental evolution. After 17 months under constant light and acetate as a carbon source, our results demonstrate that gene expression in the *C. reinhardtii* evolved population (EL) differs substantially from the progenitor population (PL). A similar experiment done with a freshwater strain of the brown seaweed *Ectocarpus* that was exposed for 6 months to seawater showed that this alga was able to adapt to the new environment. Gene expression was strongly modified in the freshwater strain so that it resembled the expression pattern of a marine member of this species [35].

Analysis of RNA-seq data from the EL population showed that, by far, the greatest number of significantly up regulated genes encoded ribosomal proteins (Fig. 3A, Fig. S3). Concomitant with this emphasis on protein production in EL cells was the significant up regulation of genes involved in RNA-transport to facilitate gene expression and to support translation. These included the translation initiation factor subunits and two subunits of RNA polymerase (Table S4). These results agree with the known correlation between higher ribosomal protein content and protein translation with increased cellular growth rates [36].

The pathways significantly down regulated in the EL population included biosynthesis of secondary metabolites (33/44 gene functions), DNA replication (8/10 gene functions), and photosynthesis (e.g., 4/4 antenna proteins) with the latter likely being a response to continuous light. For the DNA replication pathway, the mini-chromosome maintenance (MCM) complex was significantly down regulated (Fig. 3B). Several cell cycle genes (e.g., SCF, CycA, PCNA) were up regulated as would be expected in a rapidly dividing cell population (Fig. S4). The MCM complex is a helicase crucial to DNA replication and elongation that acts in the G_1_ phase as part of the transition from the pre-replicative (pre-RC) to the pre-initiation complex [37], [38]. In fission yeast, lowered expression of MCM complex proteins results in genome instability and DNA damage (i.e., abundant pre-RC is important for surviving replication stress that causes double-strand DNA breaks [39], [40]. Despite its key role in maintaining genome integrity under stress [41], this complex was significantly down regulated in the EL population as were the genes encoding DNA polymerase type-B alpha, DNA polymerase delta subunit one, and the PFC-clamp loader. The significant up regulation of the enzymes PCNA and RPA, that are involved in DNA replication and damage repair (Fig. 3B) may indicate that a balance was reached in EL cells between speed (due to an enhanced growth rate) and fidelity of DNA replication.

These results also must reflect the exposure to continuous light for EL cells that likely leads to loss or impairment of the circadian clock in *C. reinhardtii* that is reset by blue/green and red light [42]–[44]. To test this idea, we searched for significant gene expression differences among a number of clock-related genes in this alga [42]. These included RHYTHM OF CHLOROPLAST (ROC) genes [43], cryptochromes (*CPH1*), phototropins, phytochromes, and casein kinases (*CK1*, *CK2B*). Clock-related genes that showed significant down-regulation were *CPH1* that encodes a cryptochrome related to DNA photolyases that act as photoreceptors degraded in light [45]. A serine/threonine-kinase domain-containing proteins homologous to casein kinase (*CK2B*) also showed significant down-regulation, whereas another casein kinase associated with the flagellar proteome (*CK1*) [46] showed significant up-regulation (Table S4). Both of these latter genes putatively phosphorylate components of the circadian clock. Therefore, the impact of long-term exposure to continuous light in EL cells likely has complex impacts on the circadian clock in the alga that are yet to be fully understood. In addition, high light stress can lead directly to DNA damage in *C. reinhardtii* [47] although the EL cells were not exposed to high light and if so, this would presumably lead to up regulation of genes encoding the MCM complex.

Given the key role of acetate in EL growth enhancement, we investigated the metabolism of this organic carbon source in *C. reinhardtii*. Acetate can be incorporated into acetyl coenzyme A in two ways in this alga, both of which require ATP. The first is through the direct action of acetyl-CoA synthetase and the second is *via* a two-step reaction that involves acetate kinase and phosphate acetyltransferase (e.g., [48], [49]). Whereas the latter reaction does not show significant differential regulation in the EL cells, we find a 9-fold increase in the mRNA abundance of cytosolic acetyl-CoA synthetase (Table S4), suggesting this is the major route of acetate assimilation. Acetyl-CoA is fed into the respiratory tricarboxylic acid (TCA) cycle in the mitochondrion, producing NADH and assimilatory metabolic precursors or enters the glyoxylate cycle resulting in the net assimilation of carbon [11], [48], [49]. Acetyl-CoA can also enter the plastid to support lipid biosynthesis, particularly under nitrogen-limiting conditions [50], [51]. In the light, the energy required for acetate assimilation is derived from cyclic phosphorylation from photosystem I [49]. Given this existing knowledge, our data are broadly consistent with previous observations showing that heterotrophic growth leads to down regulation of photosystems [52], [53]. Transcripts encoding light harvesting chlorophyll a/b binding proteins, photosystem reaction center proteins, and plastid [Mn] superoxide dismutase (MSD3; [54]) were significantly down regulated, suggesting that the energy required for acetate assimilation likely comes from respiration. However, the mRNA abundance encoding different isoforms of enzymes in the TCA and glyoxylate cycles displayed a mixed pattern of change (Table S4) making it difficult to ascertain the flow of carbon through these pathways. Evidence for energy generation through oxidative phosphorylation is provided by the significant up regulation of genes encoding components of the respiratory complexes such as *NUOB12*, *COX90*, *PET191*, *QCR* and *QCR9* (Table S4). Cellular respiration requires Fe-S clusters and heme and produces reactive oxygen species (ROS). The EL population showed up regulation of genes involved in mitochondrial Fe-S cluster biogenesis (e.g., *ISC1* and *ISU1*) and iron transport (*FTR1*) and mitochondrial ROS metabolism (*GPX3*, *GPX5*, *TRXo*). Collectively, our results suggest that acetyl-CoA is likely to be both assimilated and respired.

**Figure 5:**
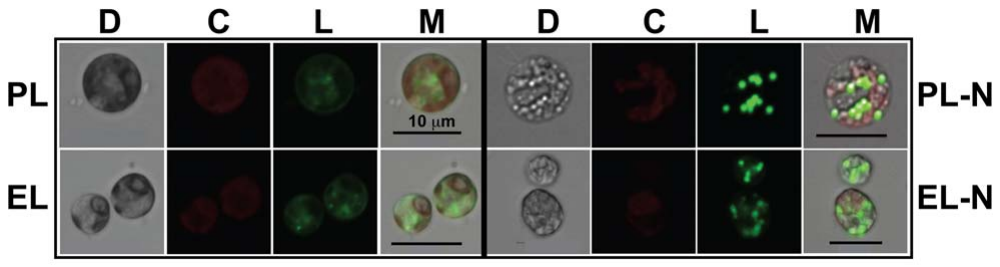
Analysis of lipid production in the PL and EL populations of *C. reinhardtii* when grown under continuous light in liquid TAP medium. Shown are differential interference contrast images (D), chlorophyll autofluorescence (C), lipid bodies (Bodipy stain; L), and merged (M). Note that like the PL and EL cells show up regulation of lipid production after depletion of nitrogen (-N) in the medium.

The variety of significant gene expression differences found in the PL-EL comparison is summarized in Figure 4. Of particular interest is the impact of selection on lipid metabolism that is a key target for biofuel strain improvement. Our analysis (Fig. S5) shows that fatty acid biosynthesis (i.e., the precursors of storage lipids) is significantly down regulated in the EL population (i.e., *fabD* and *fabH* that are involved in fatty acid initiation and elongation) under the constraint of a high growth rate. Given this result, we tested whether fast growing EL cells could be induced to produce lipids by reducing nitrogen in the medium, as has been shown for many other algae [50], [51]. Staining of the PL and EL populations with the neutral lipid dye Bodipy 493/503 [23] reveals the accumulation of lipids in both populations (Fig. 5). Therefore acetyl-CoA, which was primarily used to support algal growth under the nitrogen-replete condition, was relocated to the plastid under nitrogen-limitation to generate storage compounds.

## Conclusions

Our results demonstrate that selection using a serial dilution regime can be used to substantially modify gene expression patterns in *C. reinhardtii*. In the case of the faster growing EL population, these cells can be manipulated to produce neutral lipids, which is of interest to industry (e.g., [55]). The significant growth enhancement in *C. reinhardtii* was largely supported by improved acetate metabolism. This suggests that pathways of organic carbon usage present in many algae may provide useful targets for strain improvement. A large variety of DNA sequence and gene expression differences, the latter for fundamental process such as DNA replication and protein translation were uncovered in the PL-EL comparison. Many questions however still remain about our results such as the basis of the expression modification: e.g., is the phenotype permanent or does it reflect long-term acclimation due to epigenetic modification that can be reversed over time? We also do not know yet the reproducibility of the EL phenotype and if its underlying causes would be recapitulated in independent cultures using the same treatment. These questions are the targets of ongoing research in our lab. Nevertheless, our results clearly demonstrate the enormous capacity of algal genomes to adapt to changing conditions, a feature that can be exploited to advance basic and applied research in microbial eukaryotes.

## Supporting Information

Figure S1 **Analysis of** ***C. reinhardtii*** **RNA-seq data.** (A) Principal components analysis of gene expression for the triplicate RNA-seq experiments done with the PL and EL populations of *C. reinhardtii*. These results demonstrate that the transcriptome data derived from both sets of triplicate cultures provided a consistent signal of gene expression. (B) Expression differences using RNA-seq data from the PL-EL comparison. The Log_2_ fold changes for points shown in red are significantly different (p-value <0.05 with FDR correction) from the mean of normalized counts in that experiment.
(PDF)

Figure S2 **Results of qPCR analysis of three genes that were expressed in the PL and EL populations.** Mitochondrial cytochrome c oxidase (COX12) was used as the control in this experiment. The error bars for the qPCR analysis represent the error associated with biological triplicate measurements using RNA from the PL-EL populations. The genes names are as follows: ribosomal protein L23a, ribosomal protein L35, and DNA-directed RNA polymerase II.
(PDF)

Figure S3 **KEGG pathway analysis shows that in the PL-EL comparison, 29/30 significantly differentially expressed ribosomal proteins identified in our RNA-seq data were up regulated in the EL population.** In this figure, the background non-differentially expressed proteins are shown in light blue, whereas those that are up or down-regulated are shown in red and dark blue, respectively.
(PDF)

Figure S4 **KEGG pathway analysis of cell cycle proteins in the PL-EL comparison shows that many cell cycle genes are up regulated, whereas DNA replication (e.g., ORC, origin recognition complex) and repair (e.g., MCM complex) genes are significantly down regulated.** In this figure, the background non-differentially expressed proteins are shown in the light blue boxes, whereas those that are significantly up or down-regulated are shown in red and dark blue boxes, respectively.
(PDF)

Figure S5 **KEGG pathway analysis of genes that are significantly up or down-regulated in the pathways of fatty acid biosynthesis, metabolism, and elongation in the PL-EL comparison.** In this figure, the background non-differentially expressed proteins are shown in the light blue boxes, whereas genes that are down regulated are shown in the dark blue boxes.
(PDF)

Table S1 **Summary of the genome and transcriptome data generated for this study.**
(DOCX)

Table S2 **List of genes and primers used for the qPCR analysis.**
(DOCX)

Table S3 **Distribution of single nucleotide polymorphisms (SNPs) and insertions-deletions (indels) in the comparison of DNA sequenced from the EL and PL populations and analysis of mutation rates in the EL cells.**
(XLSX)

Table S4 **Results of RNA-seq analysis of the PL-EL populations of** ***C. reinhardtii*** **.**
(XLSX)
