# Supplementary material for: Using Natural Selection to Explore the Adaptive Potential of Chlamydomonas reinhardtii
Source: PLoS One. 2014 Mar 21;9(3):e92533. doi: 10.1371/journal.pone.0092533 (PMC3962425; doi:10.1371/journal.pone.0092533)
Supplement: Figure S1 — Analysis of C. reinhardtii RNA-seq data. (A) Principal components analysis of gene expression for the triplicate RNA-seq experiments done with the PL and EL populations of C. reinhardtii. These results demonstrate that the transcriptome data derived from both sets of triplicate cultures provided a consistent signal of gene expression. (B) Expression differences using RNA-seq data from the PL-EL comparison. The Log2 fold changes for points shown in red are significantly different (p-value <0.05 with FDR correction) from the mean of normalized counts in that experiment. (PDF) [file pone.0092533.s001.pdf]

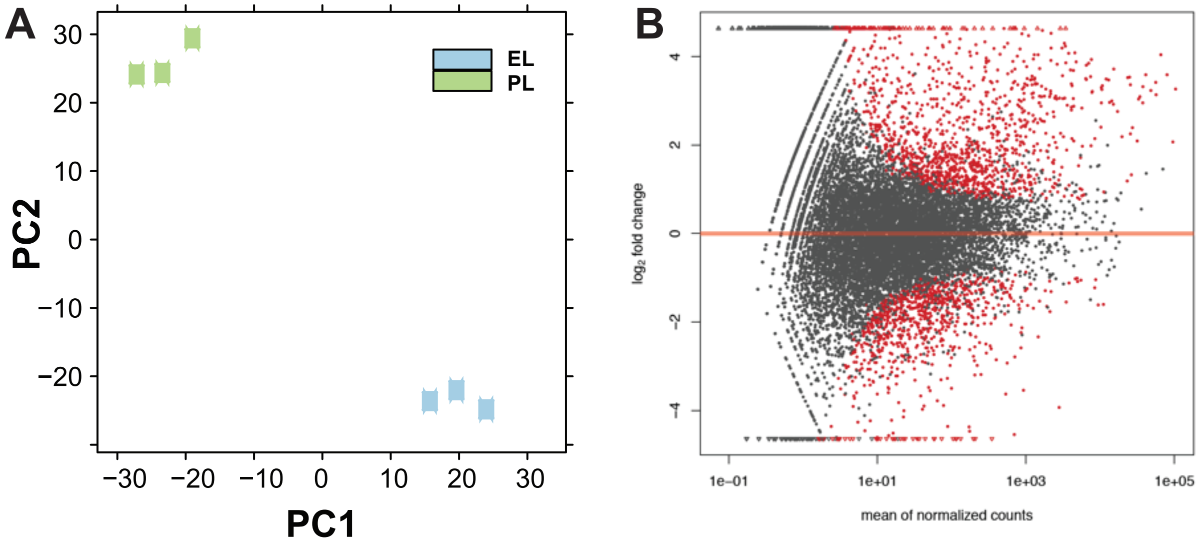

Figure S1. Analysis of *C. reinhardtii* mRNA-seq data. (A) Principal components analysis of gene expression for the triplicate mRNA-seq experiments done with the PL and EL populations of *C. reinhardtii*. These results demonstrate that the transcriptome data derived from both sets of triplicate cultures provided a consistent signal of gene expression. (B) Expression differences using mRNA-seq data from the PL-EL comparison. The Log2 fold changes for points shown in red are significantly different (*p*-value < 0.05 with FDR correction) from the mean of normalized counts in that experiment.
