# Supplementary material for: Using Natural Selection to Explore the Adaptive Potential of Chlamydomonas reinhardtii
Source: PLoS One. 2014 Mar 21;9(3):e92533. doi: 10.1371/journal.pone.0092533 (PMC3962425; doi:10.1371/journal.pone.0092533)
Supplement: Figure S2 — Results of qPCR analysis of three genes that were expressed in the PL and EL populations. Mitochondrial cytochrome c oxidase (COX12) was used as the control in this experiment. The error bars for the qPCR analysis represent the error associated with biological triplicate measurements using RNA from the PL-EL populations. The genes names are as follows: ribosomal protein L23a, ribosomal protein L35, and DNA-directed RNA polymerase II. (PDF) [file pone.0092533.s002.pdf]

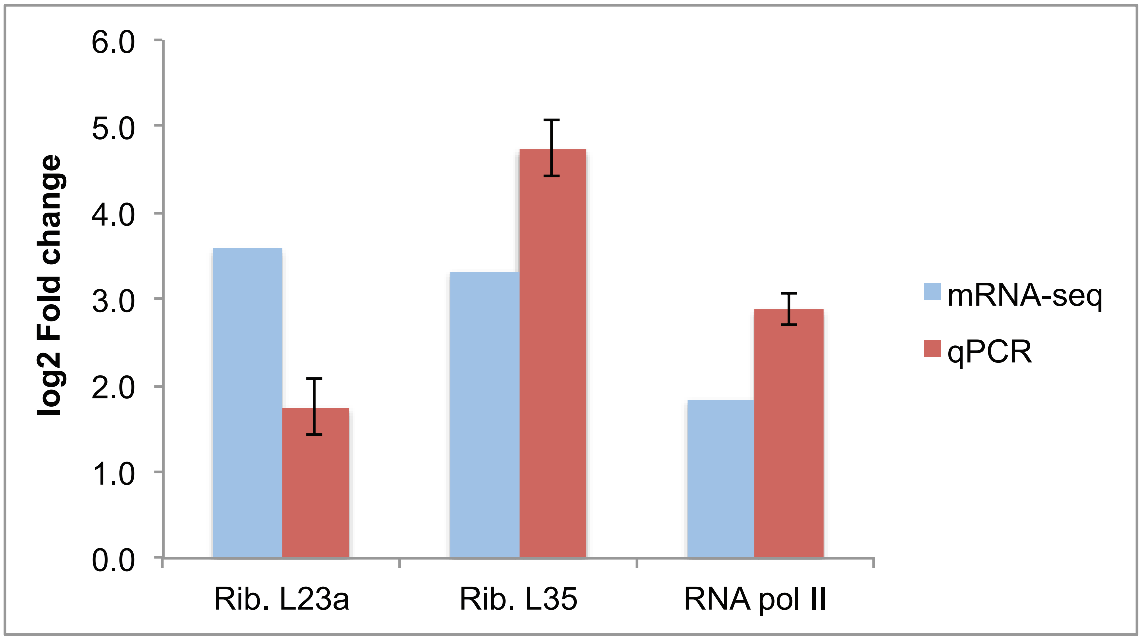

Figure S2. Results of qPCR analysis of three genes that were expressed in the PL and EL populations. Mitochondrial cytochrome c oxidase (COX12) was used as the control in this experiment. The error bars for the qPCR analysis represent the the error associated with biological triplicate measurements using RNA from the PL-EL populations. The gene names are are as folows: ribosomal protein L23a, ribosomal protein L35, and DNA-directed RNA polymerase II.
