# Supplementary material for: Using Natural Selection to Explore the Adaptive Potential of Chlamydomonas reinhardtii
Source: PLoS One. 2014 Mar 21;9(3):e92533. doi: 10.1371/journal.pone.0092533 (PMC3962425; doi:10.1371/journal.pone.0092533)
Supplement: Figure S3 — KEGG pathway analysis shows that in the PL-EL comparison, 29/30 significantly differentially expressed ribosomal proteins identified in our RNA-seq data were up regulated in the EL population. In this figure, the background non-differentially expressed proteins are shown in light blue, whereas those that are up or down-regulated are shown in red and dark blue, respectively. (PDF) [file pone.0092533.s003.pdf]

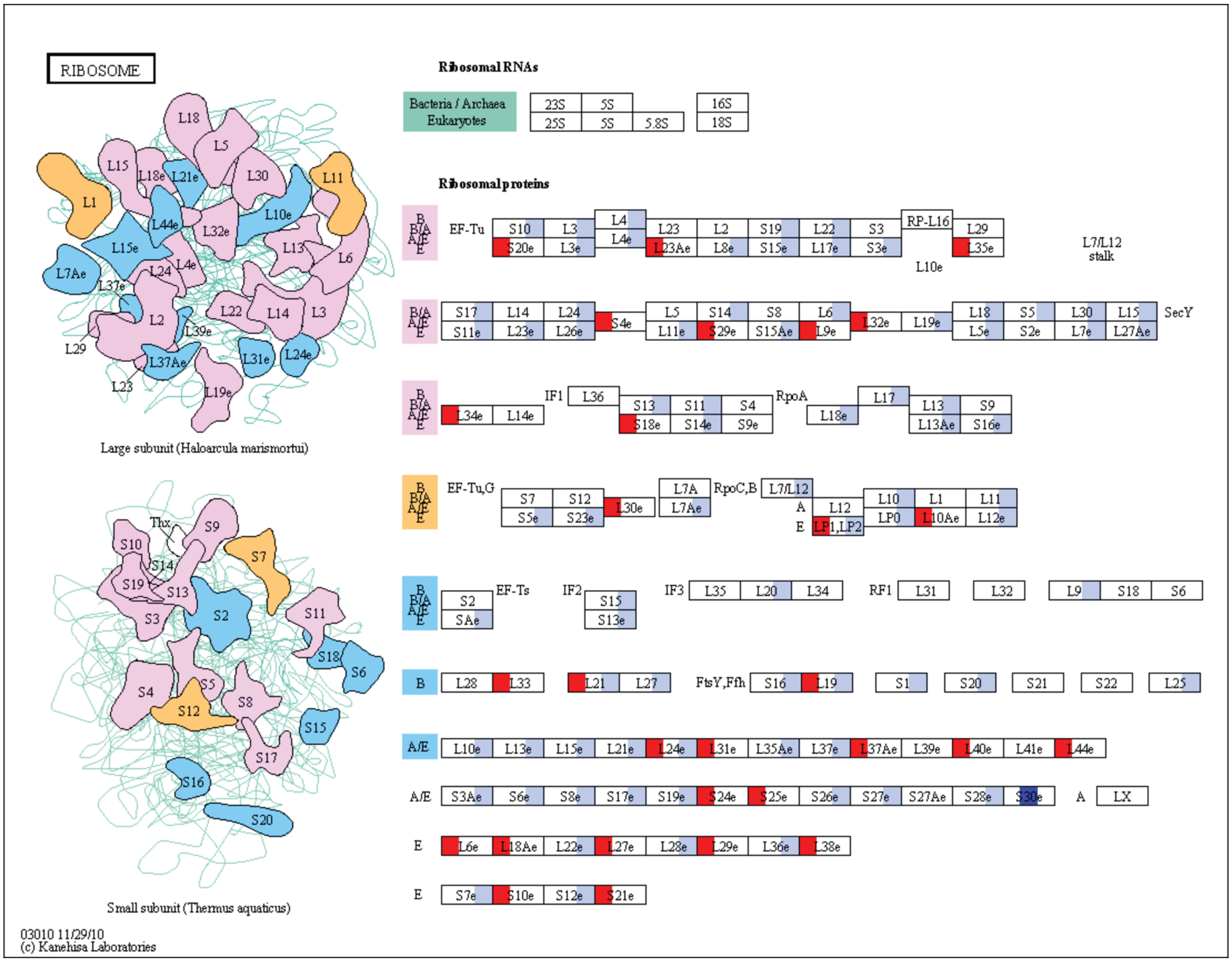

Figure S3. KEGG pathway analysis shows that in the PL-EL comparison, 29/30 significantly differentially expressed ribosomal proteins identified in our mRNA-seq data were up-regulated in the EL population. In this figure, the background non-differentially expressed proteins are shown in light blue, whereas those that are up- or down-regulated are shown in red and dark blue, respectively.
