# Supplementary material for: Using Natural Selection to Explore the Adaptive Potential of Chlamydomonas reinhardtii
Source: PLoS One. 2014 Mar 21;9(3):e92533. doi: 10.1371/journal.pone.0092533 (PMC3962425; doi:10.1371/journal.pone.0092533)
Supplement: Figure S4 — KEGG pathway analysis of cell cycle proteins in the PL-EL comparison shows that many cell cycle genes are up regulated, whereas DNA replication (e.g., ORC, origin recognition complex) and repair (e.g., MCM complex) genes are significantly down regulated. In this figure, the background non-differentially expressed proteins are shown in the light blue boxes, whereas those that are significantly up or down-regulated are shown in red and dark blue boxes, respectively. (PDF) [file pone.0092533.s004.pdf]

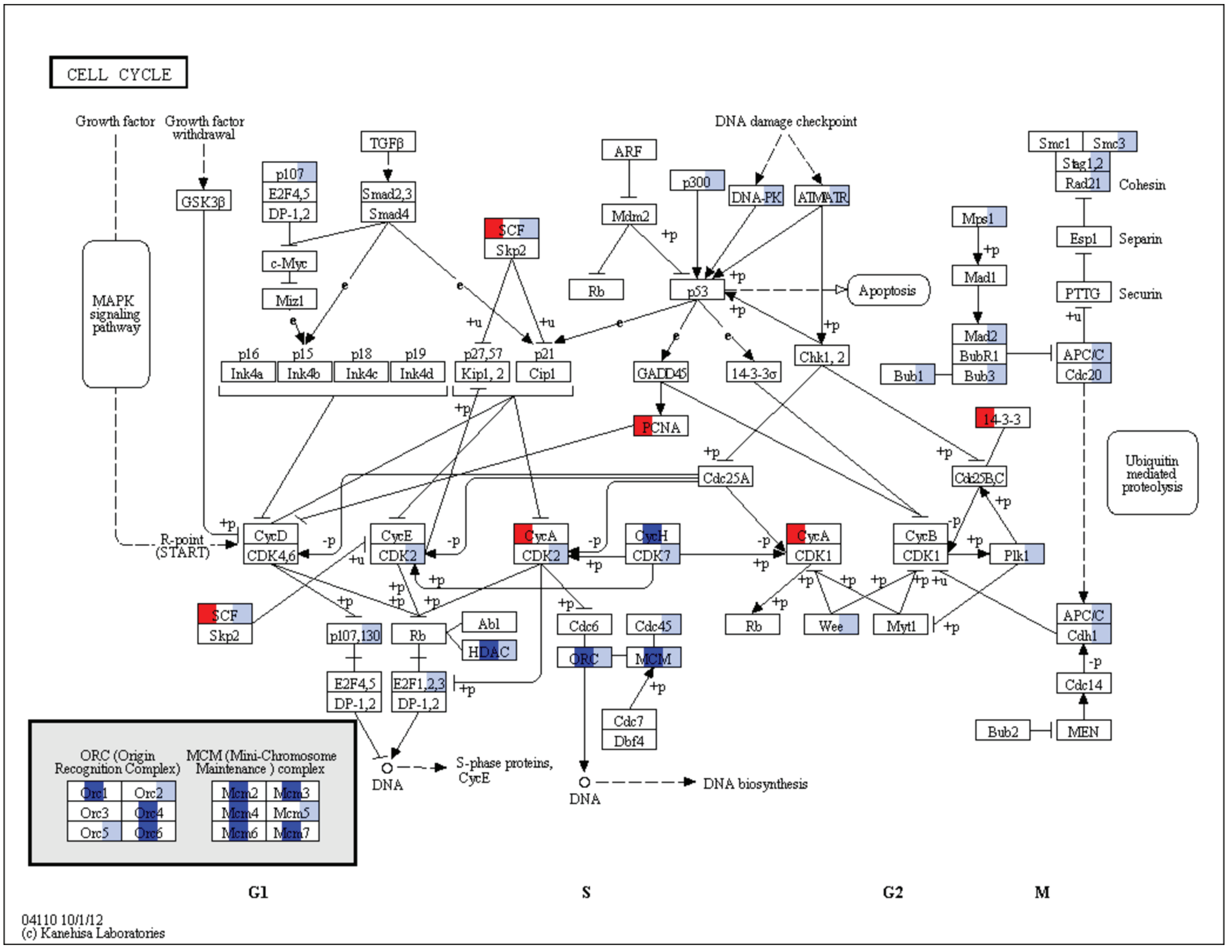


**Figure S4.** KEGG pathway analysis of cell cycle proteins in the PL-EL comparison shows that many cell cycle genes are up-regulated, whereas DNA replication (e.g., ORC, origin recognition complex) and repair (e.g., MCM complex) genes are significantly down regulated. In this figure, the background non-differentially expressed proteins are shown in the light blue boxes, whereas those that are significantly up- or down-regulated are shown in red and dark blue boxes, respectively.
