# Supplementary material for: Using Natural Selection to Explore the Adaptive Potential of Chlamydomonas reinhardtii
Source: PLoS One. 2014 Mar 21;9(3):e92533. doi: 10.1371/journal.pone.0092533 (PMC3962425; doi:10.1371/journal.pone.0092533)
Supplement: Table S1 — Summary of the genome and transcriptome data generated for this study. (DOCX) [file pone.0092533.s006.docx]

**Table S1.** Summary of the genome and transcriptome data generated for this study. The number of reads represents independent pieces of data from the sequencing machine even if they are derived from a single fragment. Therefore, 30 million reads from a paired-end library represent 15 million amplicons.

|  |  | **Length of reads** | **Number of reads** |
| --- | --- | --- | --- |
| **Genome** | Progenitor | 300x300 bp (MiSeq) | 16,012,456 |
|  | Evolved Light | 150x150 bp (MiSeq) | 14,547,343 |
|  |  | 150x150 bp (GAIIx) | 44,669,893 |
| **Transcriptome** | Progenitor Light | 51 bp (MiSeq) | 1,283,769 |
|  |  |  | 1,948,170 |
|  |  |  | 2,324,717 |
|  | Evolved Light | 150x150 bp (MiSeq) | 16,245,238 |
|  |  |  | 16,319,911 |
|  |  |  | 20,333,708 |
