# Supplementary material for: Using Natural Selection to Explore the Adaptive Potential of Chlamydomonas reinhardtii
Source: PLoS One. 2014 Mar 21;9(3):e92533. doi: 10.1371/journal.pone.0092533 (PMC3962425; doi:10.1371/journal.pone.0092533)
Supplement: Table S2 — List of genes and primers used for the qPCR analysis. (DOCX) [file pone.0092533.s007.docx]

**Table S2.** List of genes and primers used for the qPCR analysis.

| **Gene name** | **Gene Accession** | **FORWARD** | **REVERSE** | **Amplicon Size** |
| --- | --- | --- | --- | --- |
| Mitochondrial cytochrome c oxidase subunit | Cre06.g304350.t1.2 | CACTGCTTTGTGCGATTCAA | ACAGCGACTGGTATGCCTTC | 100 |
| Ribosomal protein L23a | Cre06.g282500.t1.2 | TAGCTTCAAGAAGAGCCGGAAG | CGCGTACTGGTCAAGCTTTTG | 130 |
| Ribosomal protein L35 | Cre14.g617900.t1.2 | AGCAAGCAGGACCTGATTACTC | ATCTTCGACAGCTTGTTGGG | 104 |
| DNA-directed RNA polymerase II | Cre13.g565550.t1.2 | ATACCATTGGCAACCTGCTG | TCTGCACCTTGACCACCATC | 111 |
