# Supplementary material for: Using Natural Selection to Explore the Adaptive Potential of Chlamydomonas reinhardtii
Source: PLoS One. 2014 Mar 21;9(3):e92533. doi: 10.1371/journal.pone.0092533 (PMC3962425; doi:10.1371/journal.pone.0092533)
Supplement: Figure S5 — KEGG pathway analysis of genes that are significantly up or down-regulated in the pathways of fatty acid biosynthesis, metabolism, and elongation in the PL-EL comparison. In this figure, the background non-differentially expressed proteins are shown in the light blue boxes, whereas genes that are down regulated are shown in the dark blue boxes. (PDF) [file pone.0092533.s005.pdf]

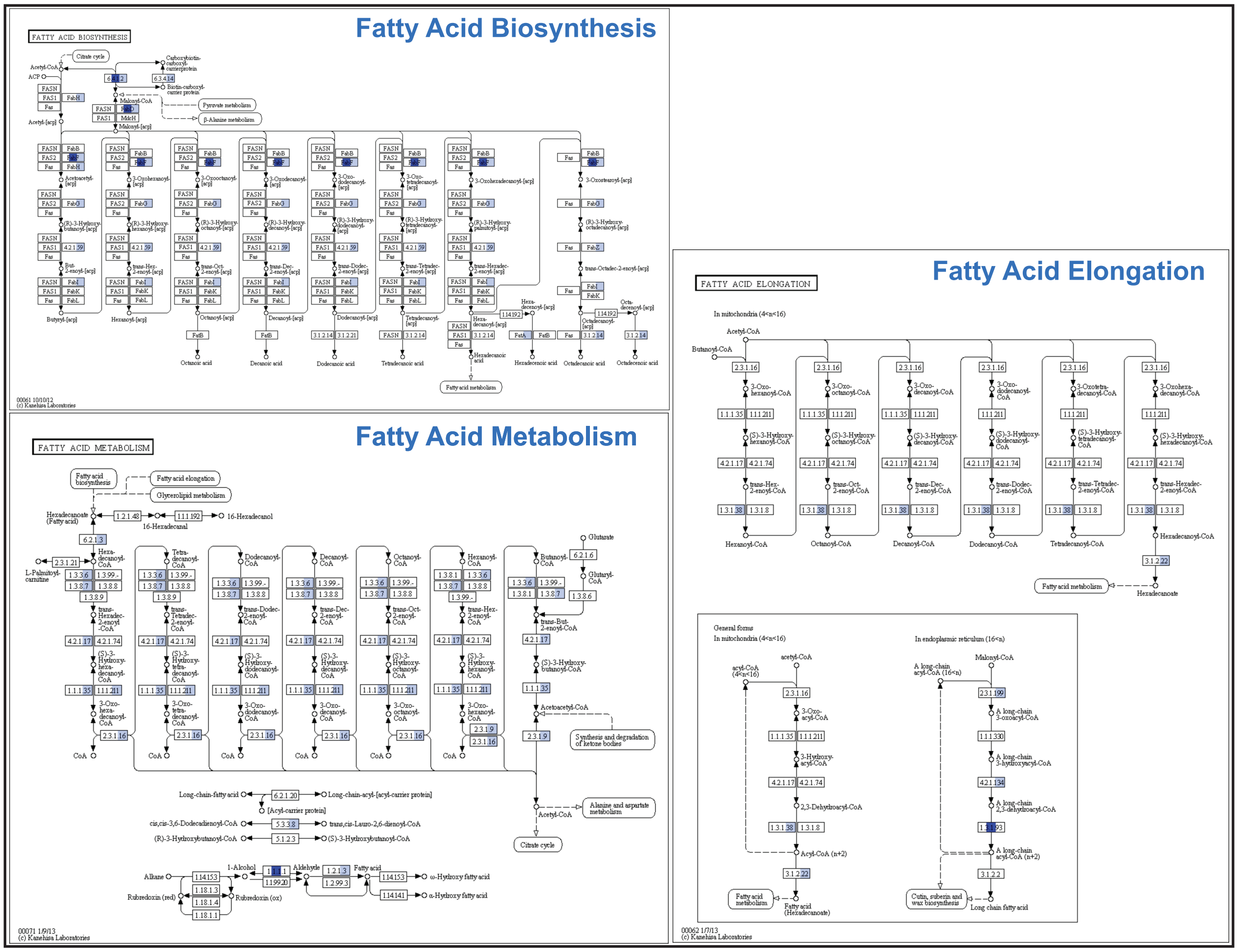

Figure S5. KEGG pathway analysis of genes that are significantly up- or down-regulated in the pathways of fatty acid biosynthesis, metabolism, and elongation in the PL-EL comparison. In this figure, the background non-differentially expressed proteins are shown in the light blue boxes, whereas genes that are down-regulated are shown in the dark blue boxes.
